# Modulation of Mn^3+^ Spin State by Guest Molecule Inclusion

**DOI:** 10.3390/molecules25235603

**Published:** 2020-11-28

**Authors:** Irina A. Kühne, Kane Esien, Laurence C. Gavin, Helge Müller-Bunz, Solveig Felton, Grace G. Morgan

**Affiliations:** 1 School of Chemistry, University College Dublin (UCD), Belfield, D04 V1W8 Dublin, Ireland; irina.kuhne@ucd.ie (I.A.K.); laurence.c.gavin@ucd.ie (L.C.G.); helge.muellerbunz@ucd.ie (H.M.-B.); 2 School of Mathematics and Physics, Queen’s University Belfast, Belfast BT7 1NN, UK; K.Esien@qub.ac.uk (K.E.); S.Felton@qub.ac.uk (S.F.)

**Keywords:** spin crossover, Mn^3+^, Schiff base, hexadentate, supramolecular, guest inclusion

## Abstract

Spin state preferences for a cationic Mn^3+^ chelate complex in four different crystal lattices are investigated by crystallography and SQUID magnetometry. The [MnL_1_]^+^ complex cation was prepared by complexation of Mn^3+^ to the Schiff base chelate formed from condensation of 4-methoxysalicylaldehyde and 1,2-bis(3-aminopropylamino)ethane. The cation was crystallized separately with three polyatomic counterions and in one case was found to cocrystallize with a percentage of unreacted 4-methoxysalicylaldehyde starting material. The spin state preferences of the four resultant complexes [MnL_1_]CF_3_SO_3_·xH_2_O, (**1**), [MnL_1_]PF_6_·xH_2_O, (**2**), [MnL_1_]PF_6_·xsal·xH_2_O, (**2b**), and [MnL_1_]BPh_4_, (**3**), were dependent on their ability to form strong intermolecular interactions. Complexes (**1**) and (**2**), which formed hydrogen bonds between [MnL_1_]^+^, lattice water and in one case also with counterion, showed an incomplete thermal spin crossover over the temperature range 5–300 K. In contrast, complex (**3**) with the BPh_4_^−^, counterion and no lattice water, was locked into the high spin state over the same temperature range, as was complex (**2b**), where inclusion of the 4-methoxysalicylaldehyde guest blocked the H-bonding interaction.

Academic Editor: Sergey G. Ovchinnikov

## 1. Introduction

Crystalline forms of spin crossover (SCO) transition metal complexes are well known to be sensitive to lattice contents, and crystal engineering can be used to a good effect to modulate the thermal evolution pathway. This has been convincingly demonstrated in mononuclear complexes of Fe^2+^ [1,2,3,4,5,6], Fe^3+^ [7,8,9,10] and Co^2+^ [11,12,13,14] and with SCO 1-D [15,16,17,18], 2-D [19,20,21,22,23] and 3-D [24,25,26,27] polymeric networks. Intermolecular interactions including hydrogen bonding [28,29,30] and π–π stacking [16,31,32,33,34,35] can be used to connect SCO-active sites and to change internal lattice pressure. Hydrogen-bonding is a particularly effective tool in modulating SCO and this approach was successfully used to generate an extended homochiral 2D sheet of mixed valence Fe^2+^/Fe^3+^ sites, which exhibited SCO and light induced excited spin state trapping (LIESST) [36]. In our own work with the [Mn(R-sal_2_323)]^+^ series of Schiff base complexes, we have established that thermal SCO is possible in the less studied *d^4^* ion Mn^3+^ [37,38,39,40,41,42,43,44,45,46]. The hexadentate ligand type used in this approach results from condensation of 1,2-bis(3-aminopropylamino)ethane with a substituted 2-hydroxybenzaldehyde, and the ligand series may be abbreviated as R-Sal_2_323 to indicate the 323 alkyl connectivity in the starting tetraamine and the substitution (R) on the phenolate ring. Changing lattice solvation and/or the identity of the polyatomic charge-balancing counterion X^−^ in the [Mn(R-sal_2_323)]X complex type has enabled examination of the effect of crystal engineering on the choice of the spin state and has yielded a variety of thermal evolution profiles [38,41,42,44]. Recently we have determined that use of the more sterically demanding naphtholate donor in place of a substituted phenolate stabilizes the rare spin triplet form of Mn^3+^ up to room temperature in crystalline samples, regardless of choice of counterion.[43] We also showed that in the naphtholate donor series, introduction of ethanol solvate guest molecules to the lattice changes the preferred electronic configuration of the Mn^3+^ to the spin quintet form, and in neither case (spin triplet or spin quintet) did we observe thermal SCO [43]. This latter result nicely demonstrates the delicate interplay between lattice contents and choice of spin paired or unpaired arrangement in Mn^3+^ and we build on this result now to demonstrate the effect of introducing a larger guest molecule. This was achieved by the serendipitous cocrystallization of some unreacted starting aldehyde during preparation of [Mn(4-methoxy-sal_2_323)]^+^ complexes with a variety of counterions designed to help (CF_3_SO_3_^−^, PF_6_^−^) or hinder (BPh_4_^−^) hydrogen-bond formation. A magnetostructural study of the resultant complexes [MnL_1_]CF_3_SO_3_·xH_2_O, (**1**), [MnL_1_]PF_6_·xH_2_O, (**2**), [MnL_1_]PF_6_·xsal·xH_2_O, (**2b**), and [MnL_1_]BPh_4_, (**3**), enabled comparison of the effect of damping intermolecular interactions by the use of a counterion ion where there is no potential for hydrogen bonding (BPh_4_^−^), with the effect of increasing the distance between SCO sites by inclusion of a guest molecule.

## 2. Results

### 2.1. Synthetic Approach

**Scheme 1:**
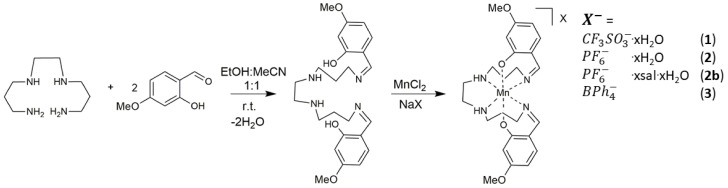
Synthesis of [MnL_1_]X complexes (**1**)–(**3**).

The reaction of 4-methoxysalicylaldehyde in a 2:1 ratio with 1,2-bis(3-aminopropylamino)-ethane, led to the formation of the Schiff base ligand H_2_L_1_, which is a suitable hexadentate ligand to chelate a Mn^3+^ centre. This resulted in the formation of dark red/black crystals of complexes (**1**)–(**3**), which were prepared in a one-pot synthesis, Scheme 1. The structures of all compounds were established by single crystal X-ray diffraction and the bulk samples were then fully characterized using elemental analysis, IR spectroscopy and magnetic measurements. Complexes (**1**)–(**3**) were formed by a salt metathesis procedure with the target counterions introduced as their group 1 salts, for example sodium as indicated in Scheme 1. This method led to the successful crystallization of [Mn(4-methoxy-sal_2_323)]^+^ (hereafter termed [MnL_1_]^+^) in the preferred crystal lattice. The use of the triflate salt led to the formation of hydrated species [MnL_1_]CF_3_SO_3_·0.7H_2_O (**1**) while the use of the larger, non-hydrogen-bonding tetraphenylborate salt yielded [MnL_1_]BPh_4_ (**3**). Salt metathesis with hexafluorophosphate yielded [MnL_1_]PF_6_·0.5H_2_O (**2**) and its inclusion complex [MnL_1_]PF_6_·xsal·xH_2_O, (**2b**) in two separate preparation attempts. In the case of complexes (**1**), (**2**) and (**2b**) the fraction of solvation/guest molecule present in the bulk samples used for magnetic measurements was estimated from elemental analysis of the microcrystalline samples, see Section 4.2.

#### Synthesis and Characterization of Compounds (**1**)–(**3**)

In the case of the single crystals used in the diffraction experiments the solvent/guest could not be determined crystallographically in terms of atomic sites. Therefore, Platon SQUEEZE [47] was used to compensate for the spread electron density, leading to the respective molecular formulae listed in Table A1.

### 2.2. Magnetic Characterisation

**Figure 1:**
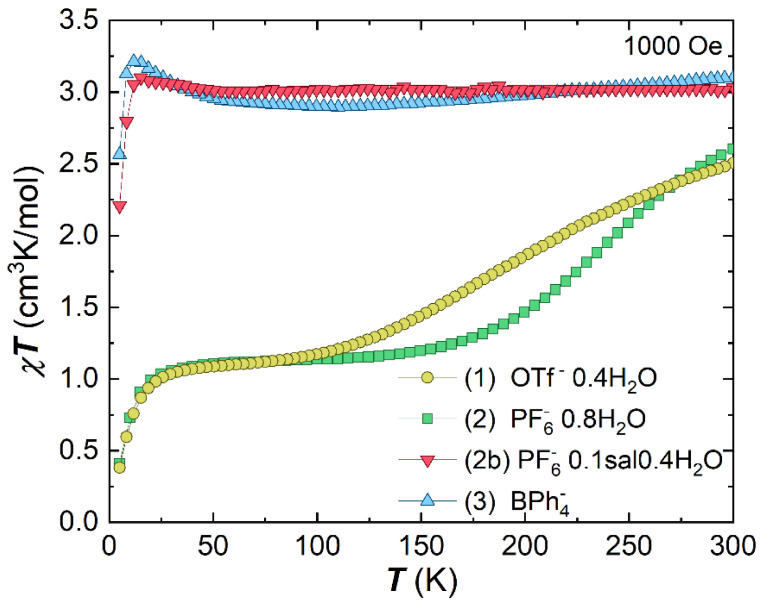
Plots of χ_M_*T* versus T for complexes (**1**) (yellow), (**2**) (green), (**2b**) (red) and (**3**) (blue) in the temperature range 5–300 K in the cooling mode.

The magnetic susceptibility of the bulk samples of compounds (**1**)–(**3**) was measured using a SQUID magnetometer and the data were collected from 300 K down to 5 K under an applied dc field of 1000 Oe, Figure 1. No thermal hysteresis was detected on warming back to room temperature and plots of χ_M_*T* versus T are shown in the cooling mode only, Figure 1.

Both hydrated compounds (**1**) and (**2**) exhibited an incomplete thermal spin transition. Below 100 K both exist in the low spin state with χ_M_*T* values close to the expected spin-only value of 1.0 cm^3^·K/mol for S = 1, assuming g = 2. Above 100 K both show SCO upon warming, with a gentle sigmoidal pathway, although neither reached the pure high-spin state by 300 K. T_1/2_ values were determined to be 217 K for (**1**) and 242 K for (**2**). In sharp contrast both complexes (**2b**) and (**3**) were in the spin quintet form over the same temperature range with χ_M_*T* values close to the expected spin only value of 3.0 cm^3^K/mol for a monomeric Mn^3+^ complex with S = 2 and g = 2.

### 2.3. Structural Characterisation of Compounds *(**1**)–(**3**)*

**Figure 2:**
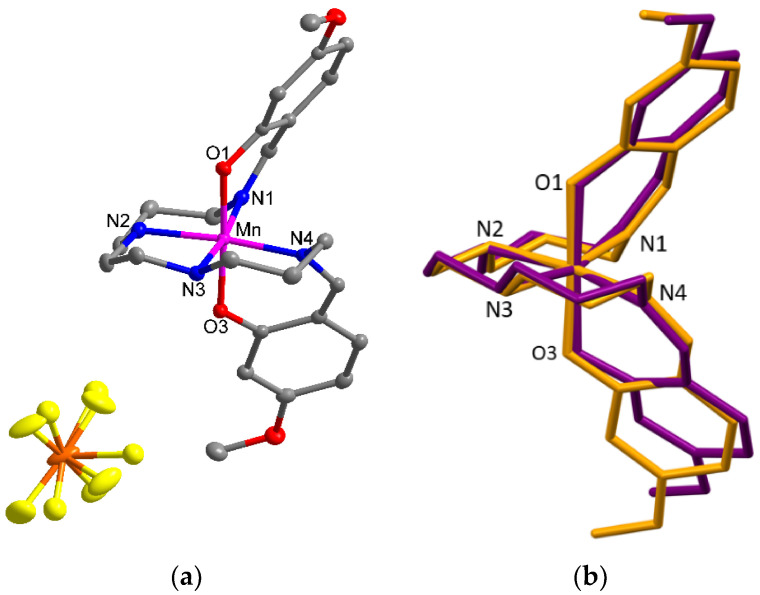
(**a**) View of complex cation and counterion in complex (**2**) at 100 K, which was modelled with one half-occupancy water molecule, [MnL_1_]PF_6_·0.5H_2_O (water molecule and hydrogen atoms omitted for clarity); (**b**) structural overlap of the cationic species of complex (**2**), [MnL_1_]PF_6_·0.5H_2_O (orange) and (**2b**), [MnL_1_]PF_6_·0.3H_2_O·0.3sal (purple) at 100 K.

Complexes (**1**) and (**2**) crystallize isostructurally in monoclinic space group *P2_1_/c* with Z = 4 where the asymmetric unit contains a full [MnL_1_]^+^ cation and one full triflate or one full hexafluorophosphate anion, respectively, Figure 2. The hexadentate Schiff base ligand chelates the Mn^3+^ centre in *pseudo* octahedral geometry with two *trans*-phenolate donors, O1 and O3, two *cis*-amine and two *cis*-imine donor atoms, in the same way as previously observed for manganese complexes with this ligand type [37,38,39,40,41,42,43,44,45,46,48]. Residual electron density in both data sets was modelled as part occupancy water molecules using Platon SQUEEZE [47]. Data for complex (**2**) was collected at 100 K and 293 K on two different crystals and the water content was modelled as half-occupancy and full occupancy respectively (Table A1).

Compound **2b**, with partial occupancy of guest molecule 4-methoxysalicylaldehyde, crystallizes in the monoclinic space group *P2_1_/n* with Z = 4. Occupancy of the lattice contents was refined with 0.3 molecules of water and 0.3 molecules of unreacted 4-methoxysalicylaldehyde, yielding a formula of [MnL_1_]PF_6_·0.3H_2_O·0.3sal for the crystalline sample. The benzene ring of the disordered salicylaldehyde was restrained to be regular using SADI and FLAT. Rigid Bond (RIGU) restraints were applied to all non-hydrogen atoms of this molecule. Note that elemental analysis of the bulk sample used in the SQUID measurements showed a better fit to [MnL_1_]PF_6_·0.4H_2_O·0.1sal so the latter formula was used in the calculation of molar magnetic susceptibility. The cell parameters of a = 17.2 Å, b = 9.6 Å and c = 19.0 Å for complex (**2b**) suggest similar dimensions to complex (**2**), (a = 8.0 Å, b = 20.8 Å and c = 16.2 Å), which was modelled as having 0.5 molecules of water in the crystal lattice. An overlay of the complex cations in (**2**) and (**2b**) is shown in Figure 2b.

Complex (**3**) crystallizes in the triclinic space group $P\bar{1}$ with Z = 2 and does not contain any guest molecules, the asymmetric unit comprises one unique [MnL_1_]^+^ cation and one well ordered full occupancy BPh_4_^−^ counterion.

In Mn^3+^ SCO compounds of the [Mn(R-sal_2_323)]^+^ type, the average bond lengths change upon spin transition, but only significantly for the amine and imine bonds in the equatorial positions. The Mn-N_imine_ bond lengths are typically 1.95–2.00 Å in the spin triplet form, increasing to 2.10–2.15 Å in the spin quintet state, while the Mn-N_amine_ bond lengths change from 2.00–2.10 to 2.20–2.30 Å during a spin transition [41]. The bond lengths of complexes (**1**)–(**3**) (Table 1) indicate that the SCO complexes (**1**) and (**2**) had typical low spin bond lengths at 100 K while high spin complexes (**2b**) and (**3**) had those typical for the S = 2 spin state. Upon warming, the bond lengths of complexes (**1**) and (**2**) show the expected equatorial elongation (Table 1) indicative of the transition towards the high spin state. The room temperature bond lengths of complexes (**1**) and (**2**) show that both compounds have not yet fully reached the pure spin quintet state, which is in good agreement with the magnetic data. This behaviour has also been observed in other Mn^3+^ SCO compounds, which show a gradual and incomplete thermal crossover [37,44], whereas the full transition to high-spin almost always leads to Mn-N_amine_ bond lengths above 2.2 Å [40,42].

In complexes (**1**) and (**2**) the increase in bond lengths in the equatorial plane led to a displacement of the benzene ring, which can be seen in the view of the overlaid structures in Figure 2b where the structural differences between the low spin compound (**2**) and the high-spin compound (**2b**) are highlighted. While most of the flexible backbone part of the Schiff base ligand overlapped almost perfectly, there were discrepancies visible in the benzene rings and the peripheral methoxy substituents.

Hexacoordinated Mn^3+^ compounds exhibit a stronger distortion of the octahedral environment in the S = 2 state than in the almost perfect octahedral geometry observed in the S = 1 state due to the Jahn-Teller effect in the high spin form. The degree of distortion, analysed by the distortion parameters Σ and Θ as defined by McKee et al. [49] highlights the local angular deviation from the *cis* octahedral angles of 90°, while Θ measures the trigonal torsion, which is defined as the degree of twist from a perfect octahedron towards trigonal prismatic geometry. Both values are zero in the case of a perfect octahedron, and the reported literature values for Mn^3+^ compounds are shown in Table 2. [41,44].

Σ and Θ have been calculated using OctaDist 2.6.1 [50] and the observed parameters are summarized in Table 3, which reflect the structural distortion due to the different spin states of the molecules. At 100 K, compounds (**1**) and (**2**) clearly exhibit Σ and Θ values which confirm that both are in the low spin state at this temperature. Upon warming, the values of both complexes increase slightly to Σ = 45.3° for (**1**) (46.3° for (**2**)) and Θ = 140.3° for **1** (145.7° for (**2**)), which reflected the gradual spin state change within these compounds. In contrast, analysis of the distortion in compounds (**2b**) and (**3**), which were determined by magnetometry to be high-spin over the whole measured temperature range, had high Σ and Θ values, confirming the S = 2 assignment. Even though the angular distortion parameter, Σ, is high for these latter two, the values are still within the range that was reported for other SCO Mn^3+^ compounds [41,44], whereas the Θ values of both are higher than what has been reported before. Olguin has recently summarized the Σ values of Mn^3+^ complexes of the [Mn(R-Sal-323]^+^ type, and shows that Σ values above 70° often lead to complexes that are locked in the high-spin state [48]. The fact that this is not necessarily always the case can be seen in the [Mn(3-MeO-Sal-323-]NO_3_ complex [37]. This complex has a Σ value of 70.7° in the high spin state, with Θ values of 129.8° in the low spin state and 222.3° in the high spin state, while still being able to undergo spin transition, in contrast to related complexes with lower Σ values, which are locked in the high spin state [48]. It appears that there is a threshold for the Σ values at around 70° but it is not exact, as SCO is observed in some Mn^3+^ complexes with Σ values higher than 70°. On the other hand, the Θ values may be a more reliable indicator of SCO in Mn^3+^, as it seems that for Θ values higher than 250°, the compounds are not able to undergo a spin transition and will remain in the high spin state. This demonstrates the considerable elastic flexibility of the [MnL_1_]^+^ complex type, which can accommodate bulky anions and neutral molecules in the crystal lattice, resulting in deformation of the complex cation with the possible loss of SCO behaviour.

Examination of the intermolecular arrangements in complexes (**1**)–(**3**) revealed that only the two complexes that exhibited thermal SCO, complexes (**1**) and (**2**), make strong intermolecular contacts (See Supplementary Materials Figures S1–S4). These were by way of hydrogen bonding, which in the case of triflate complex (**1**) connected the complex cation to the counterion via the guest water molecule at both 100 K and 293 K (Figures S1 and S2). In the case of the hexafluorophosphate complex, a hydrogen bond was confirmed between the complex cation and partial occupancy water molecule at both measured temperatures (Figures S3 and S4), but the nature of the interaction with the counterion was less clear, especially given the significant distortion of the PF_6_^−^ counterion in the 100 K structure. In marked contrast both high spin complexes (**2b**) and (**3**) had no intermolecular interactions with the complex cation and this likely contributed to the locking-in of the spin quintet state over the thermal range. In complex (**2b**) the partial occupancy guest water and 4-methoxysalicylaldehyde molecules were highly disordered but were distinct from the [MnL_1_]^+^ cation and PF_6_^−^ counterion, the latter two forming neither hydrogen bonds nor π–π interactions, Figure S5. Thus the [MnL_1_]^+^ spin carrier was isolated in this lattice with the large guest molecule, Figure S6, and persisted in a fixed spin state while the temperature was reduced. A similar fixed spin-state response was observed in complex (**3**) with the largest counterion, BPh_4_^−^, which was not disposed to form hydrogen bonds, and which also therefore tended to isolate the complex cation, thereby preventing SCO [41].

## 3. Discussion

A magnetostructural examination of a new series of SCO and high spin salts of the [MnL_1_]^+^, complex cation, in which L_1_ is the 4-methoxy-sal_2_323 hexadentate Schiff base ligand, revealed the delicate dependence of the complex spin state on lattice contents. This is because the geometry of the immediate coordination sphere of the Mn^3+^ ion will be subject to different degrees of local distortion depending on the identity of the lattice partners in the space between spin-labile [MnL_1_]^+^ cations. This in turn impacts on the ability of the coordination sphere to undergo the significant rearrangement, which is required in spin state switching. The lattice partners, comprising counterions, and/or guest molecules such as solvent or unreacted starting materials, will have both a different volume and a different capacity for forming intermolecular interactions. A thermal spin transition typically necessitates a large change in volume and/or distortion as the antibonding orbitals are depopulated on cooling. This is especially relevant in ions which have a strong distortion in either spin state, as is the case with the Jahn-Teller ions Mn^3+^ and Co^2+^, which have marked distortions in the high spin and low spin forms respectively [48]. The Jahn-Teller effect is also relevant in some Fe^2+^ systems [5]. In the case of the complexes reported here two factors may be at work: Firstly, the inclusion of sterically demanding lattice partners such as a guest 4-methoxysalicylaldehyde molecule or large BPh_4_^−^ counterion fills up the void space and causes a marked distortion of the coordination geometry around the Mn^3+^ ion in the high spin state. Thus, the degree of distortion in the high spin forms, as revealed by the Θ angle (Table 3), is higher in those complexes which do not show SCO but which are locked into the high spin form over the temperature range. In contrast those complexes that can undergo SCO, complexes (**1**) and (**2**), show less distortion in their high spin forms, Table 3, suggesting that there is less strain with the less sterically demanding PF_6_^−^ and CF_3_SO_3_^−^, counterions. 

Secondly it appears that hydrogen bonding plays an important role in coupling the spin labile complex cation to the lattice, and in its absence the complex can get “stuck” in one spin state, in the case of [MnL_1_]^+^ this is the spin quintet form. The connectivity of the complex cation can be modulated by choice of counterion: use of counterions with oxygens and fluorines such as PF_6_^−^ and CF_3_SO_3_^−^, enables efficient hydrogen bond formation with one of the phenoxy donors on the complex and SCO ensues. Substitution with BPh_4_^−^ rules out hydrogen bonding and there is a concomitant loss of spin state lability for the cation in this lattice. We also established here that guest molecules other than the solvent could be accommodated, and inclusion of partial occupancy 4-methoxysalicylaldehyde in one of the synthetic attempts to make the PF_6_^−^ complex, blocks the hydrogen bonding that was previously observed with this combination and the SCO is quenched. The importance of crystal engineering in modulating spin state preferences in such Mn^3+^ chelates is clear and our work on related systems continues to try to establish the extent of the phenomenon in such materials.

## 4. Materials and Methods

### 4.1. Materials and Physical Measurements

All chemicals and solvents if not otherwise mentioned were purchased from chemical companies and were reagent grade. They were used without further purification or drying. All reactions were carried out under ambient conditions. All measurements were carried out on powdered samples of the respective polycrystalline compound. Elemental analysis (C, H and N) were performed using a Perkin Elmer Vario EL (Waltham, MA, USA). A Bruker Alpha PlatinumATIR spectrometer (Billica, MA, USA), which was used to record the infrared spectra, and mass spectra were recorded on a Waters 2695 Separations Module Electrospray Spectrometer (Milford, MA, USA).

### 4.2. Synthesis and Characterisation of Compounds *(**1**)–(**3**)*

*Complex [MnL_1_]CF_3_SO_3_·0.4H_2_O* (**1**). H_2_L_1_ (4-methoxy-sal_2_323) was synthesised using 0.076 g (0.5 mmol) 4-methoxylsalicylaldehyde together with 0.044 mg (0.25 mmol) 1,2-bis(3-aminopropyl-amino)ethane, in ethanol/acetonitrile (1:1) (10.0 mL). The ligand solution was stirred for one hour under ambient conditions to complete the Schiff base reaction and was then used directly without further purification. The ligand solution of H_2_L_1_ was added by gravity filtration to a solution 0.25 mmol MnCl_2_·4H_2_O (0.046 g) dissolved in ethanol/acetonitrile (1:1) (10 mL) together with 0.3 mmol LiCF_3_CO_3_ (0.047 g). The solution turned dark red (almost black) and was stirred for 10 min at r.t. Any precipitate was filtered off afterwards and the reaction was left for slow evaporation. After a few days, small dark red-purple crystalline plates were isolated by filtration, which were suitable for single crystal X-ray analysis. (yield: 0.040 g, 6%). Mass spectrometry (g/mol): expected: 495.18 (100% complex cation); found: 495.02. Elemental analysis for **1**, [C_24_H_32_N_4_O_4_Mn]^+^[CF_3_SO_3_]^−^·0.4H_2_O (%): calculated: C: 46.07; H: 5.07; N: 8.60; S: 4.92. Found: C: 45.85; N: 4.97; N: 8.48; F: 5.14.

*Complexes [MnL_1_]PF_6_·0.8H_2_O* (**2**) *and [MnL_1_]PF_6_·0.1sal·0.4H_2_O* (**2b**). The ligand solution of H_2_L_1_ was added by gravity filtration to a solution 0.25 mmol MnCl_2_·4H_2_O (0.046 g) dissolved in ethanol/acetonitrile (1:1) (10 mL) together with 0.3 mmol KPF_6_ (0.055 g). The solution turned dark red (almost black) and was stirred for 10 min at r.t. Any precipitate was filtered off afterwards and the reaction was left for slow evaporation. After a few days, small dark red-purple thin crystalline plates were isolated by filtration. (Yield: 0.055 g, 10%). Complex [MnL_1_]PF_6_·0.1sal·0.4H_2_O (**2b**) was recovered in a separate synthesis attempt.

*Complex [MnL_1_]PF_6_·0.8H_2_O* (**2**). Mass spectrometry (g/mol): expected: 495.18 (100% complex cation); found: 495.04. Elemental analysis for (**2**), [C_24_H_32_N_4_O_4_Mn]^+^[PF_6_]^−^·0.8H_2_O (%): calculated: C: 44.02; H: 5.17; N: 8.56; F: 17.41. Found: C: 44.17; N: 5.04; N: 8.41; F: 17.38.

*Complex [MnL_1_]PF_6_·0.1sal·0.4H_2_O* (**2b**). Mass spectrometry (g/mol): expected: 495.18 (100% complex cation); found: 495.02. Elemental analysis for **2b**, [C_24_H_32_N_4_O_4_Mn]^+^[PF_6_]^−^·0.1(C_8_H_8_O_3_)·0.4H_2_O (%): calculated: C: 44.94; H: 5.11; N: 8.456. Found: C: 44.98; N: 5.07; N: 8.37.

*Complex [MnL_1_]BPh_4_* (**3**). The ligand solution of H_2_L_1_ was added by gravity filtration to a solution 0.25 mmol MnCl_2_·4H_2_O (0.046 g) dissolved in ethanol/acetonitrile (1:1) (10 mL) together with 0.3 mmol NaBPh_4_ (0.103 g). The solution turned dark red (almost black) and was stirred 10 min at r.t. Any precipitate was filtered off afterwards and the reaction was left for slow evaporation. After a few days, small dark red-purple plates were isolated by filtration. Mass spectrometry (g/mol): expected: 495.18 (100% complex cation); found: 495.02. Elemental analysis for **3**, [C_24_H_32_N_4_O_4_Mn]^+^[BC_24_H_20_]^−^ (%): calculated: C: 70.76; H: 6.43; N: 6.88. Found: C: 70.28; N: 6.38; N: 6.73.

### 4.3. Single-Crystal X-ray Structure Determination

Suitable single crystals of complexes (**1**) to (**3**) were mounted on Oxford Diffraction Supernova E diffractometer (Oxford, UK) fitted with an Atlas detector; datasets were measured using monochromatic Cu-*K*α radiation or Mo-*K*α radiation and corrected for absorption [51]. The temperature (100 K) was controlled with an Oxford Cryosystem instrument. Structures were solved by dual-space direct methods (SHELXT) [52] and refined with full-matrix least-squared procedures based on *F^2^*, using SHELXL-2016. Non-hydrogen atoms were refined with independent anisotropic displacement parameters, organic H-atoms (i.e., bonded to C) were placed in idealized positions, while the coordinates of H-atoms bonded to O were generally refined with their O-H distance restrained to 0.88 (4) Å. Within complex (**2b**), the hydrogen atoms of the water molecules could not be detected and were therefore placed in the idealized position. The benzene ring of the disordered unreacted salicylaldehyde was restrained to be regular using SADI and FLAT. Rigid Bond (RIGU) restraints were applied to all non-hydrogen atoms of this molecule.

Selected crystallographic data and structure refinements are summarized in Table A1 and crystallographic data for the structures reported in this paper were deposited with the Cambridge Crystallographic Data Centre (CCDC) as supplementary publication numbers CCDC-2042004-2042009. Copies of the data can be obtained free of charge from https://www.ccdc.cam.ac.uk/structures/.

### 4.4. Magnetic Measurements

The magnetic susceptibility measurements were recorded on a Quantum Design SQUID magnetometer MPMS-XL (San Diego, CA, USA) operating between 1.8 and 300 K. DC measurements were performed on polycrystalline samples. Each sample was wrapped in a gelatine capsule and subjected to fields in the range from 0 to 7 T. The magnetization data was collected at 100 K in order to check for ferromagnetic impurities, which were found to be absent in the samples. Diamagnetic corrections were applied to correct for contribution from the sample holder, and the inherent diamagnetism of the sample was estimated with the use of Pascal’s constants. AC measurements were carried out at frequencies between 1 and 1500 Hz.

## Figures and Tables

**Table 1:** Mn-donor bond lengths in complexes (**1**)–(**3**).

Mn-X	OTf^−^ (1)	OTf^−^ (1)	PF_6_^−^ (2)	PF_6_^−^ (2)	PF_6_^−^ (2b)	BPh_4_^−^ (3)
Temp. (K)	100	293	100	293	100	100
Mn-O_phen_	1.874	1.872	1.881	1.876	1.879	1.866
	1.894	1.885	1.885	1.879	1.881	1.876
Mn-N_imine_	1.977	2.028	1.983	2.035	2.083	2.079
	1.990	2.068	1.990	2.088	2.139	2.131
Mn-N_amine_	2.056	2.139	2.054	2.144	2.216	2.237
	2.063	2.161	2.061	2.179	2.279	2.268
Spin State	S = 1		S = 1		S = 2	S = 2

**Table 2:** Typical Angular (Σ) and Trigonal (Θ) distortion values for spin triplet and quintet forms of Mn^3+^.

Spin State	Σ	Θ
S = 1	28°–45°	79°–125°
S = 2	48°–80°	135°–230°

**Table 3:** Distortion angle parameters, Σ (angular deviation at the origin) and Θ (trigonal torsion angle) for all [MnL_1_]X complexes (**1**)–(**3**).

	OTf^−^ (1)	OTf^−^ (1)	PF_6_^−^ (2)	PF_6_^−^ (2)	PF_6_^−^ (2b)	BPh_4_^−^ (3)
Temp. (K)	100	293	100	293	100	100
Σ	30.4	45.3	30.8	46.3	72.1	71.1
Θ	87.1	140.3	85.9	145.7	274.5	256.6
Spin State	S = 1		S = 2		S = 2	S = 2

## Supplementary Materials

Figure S1: View of asymmetric unit of [MnL_1_]CF_3_SO_3_·0.7H_2_O at 100 K showing H-bonding connecting complex cation and counterion via a water molecule, Figure S2: View of asymmetric unit of [MnL_1_]CF_3_SO_3_·0.7H_2_O at 100 K showing H-bonding connecting complex cation and counterion via a water molecule, Figure S3: View of asymmetric unit of [MnL_1_]PF_6_·0.5H_2_O at 100 K showing H-bonding between phenoxide oxygen and water molecule. A close contact is formed between the water molecule and the disordered PF_6_^−^ counterion, Figure S4: View of asymmetric unit of [MnL_1_]PF_6_·0.5H_2_O at 293 K showing H-bonding between phenoxide oxygen and water molecule, Figure S5: View of asymmetric unit of [MnL_1_]PF_6_·0.3H_2_O·0.3sal at 100 K illustrating the absence of intermolecular interactions to the complex cation and showing disorder of partial occupancy 4-methoxysalicylaldehyde guest molecule and water, Figure S6: Space filling packing arrangement of [MnL_1_]PF_6_·0.3H_2_O·0.3sal at 100 K along the a-axis (left) and along the b-axis (right), Figure S7: View of asymmetric unit of [MnL_1_]BPh_4_ at 100 K illustrating the absence of intermolecular interactions.

## Appendix A

**Table A1:** Crystallographic details for complexes (**1**)–(**3**).

**Compound**	**[MnL_1_]OTf·0.7H_2_O (1)**	**[MnL_1_]OTf·0.7H_2_O (1)**	**[MnL_1_]PF_6_·0.3H_2_O·0.3sal (2b)**
Sample code	mor206 (100 K)	mor203 (293 K)	mor1106 (100 K)
Empirical formula	C_25_H_33.3_N_4_O_7.7_F_3_SMn	C_25_H_33.5_N_4_O_7.7_F_3_SMn	C_26.6_H_35.3_N_4_O_5.3_F_6_PMn
Formula weight	658.06	658.06	696.58
Temperature (K)	100(2)	293(2)	100(2)
Radiation	Mo-Kα	Mo-Kα	Cu-Kα
Crystal system	monoclinic	monoclinic	Monoclinic
Space group	*P2_1_/c*	*P2_1_/c*	*P2_1_/n*
Crystal size (mm)	0.50 × 0.30 × 0.20	0.80 × 0.40 × 0.30	0.27 × 0.20 × 0.16
*a* (Å)	8.0779(10)	8.2335(13)	17.2396(6)
*b* (Å)	20.908(3)	21.096(3)	9.6048(2)
*c* (Å)	16.371(2)	16.582(3)	19.0249(6)
*α* (°)	90	90	90
*β* (°)	97.789(2)	98.091(3)	105.501(3)
*γ* (°)	90	90	90
V (Å^3^)	2739.3(6)	2851.6(8)	3035.61(16)
Z	4	4	4
d_calc_ (g cm^−3^)	1.586	1.533	1.524
*μ* (mm^−1^)	0.634	0.610	4.770
F(000)	1366	1366	1439
Limiting indices	h = ±11, k = ±29, l = ±23	h = ±10, k = ±26, l = ±20	h = ±21, k = ±12, l = ±23
Reflect. coll./uniq.	29310/7944	24235/5593	34913/6368
R(int)	0.0301	0.0238	0.0621
Complete to Θ (%)	99.4	99.9	99.9
Data/restr./param.	7944/2/398	5593/0/389	6368/93/473
GooF on F^2^	1.040	1.053	1.037
Final R indices [I > 2σ(I)]	R_1_ = 0.00491, wR_2_ = 0.1302	R_1_ = 0.0455, wR_2_ = 0.1215	R_1_ = 0.0412, wR_2_ = 0.1119
R indices (all data)	R_1_ = 0.0585, wR_2_ = 0.1387	R_1_ = 0.0517, wR_2_ = 0.1277	R_1_ = 0.0438, wR_2_ = 0.1155
Largest diff. peak/hole (e·Å^−3^)	1.724 and −1.337	0.722 and −0.432	0.863 and −0.554
CCDC no.	2042004	2042005	2042008
**Compound**	**[MnL_1_]PF_6_·0.5H_2_O (2)**	**[MnL_1_]PF_6_·H_2_O (2)**	**[MnL_1_]BPh_4_ (3)**
Sample code	mor1152 (100 K)	mor141 (293 K)	mor428
Empirical formula	C_24_H_33.1_N_4_O_4.5_F_6_P Mn	C_24_H_34_N_4_O_5_F_6_PMn	C_48_H_52_BN_4_O_4_Mn
Formula weight	650.24	658.46	814.69
Temperature (K)	100(2)	293(2)	100(2)
Radiation	Cu-Kα	Mo-Kα	Cu-Kα
Crystal system	monoclinic	monoclinic	triclinic
Space group	*P2_1_/c*	*P2_1_/c*	$P\bar{1}$
Crystal size (mm)	0.235 × 0.136 × 0.063	1.20 × 0.20 × 0.20	0.211 × 0.161 × 0.040
*a* (Å)	8.00626(4)	8.1783(9)	12.3155(4)
*b* (Å)	20.81514(9)	20.882(2)	14.1292(4)
*c* (Å)	16.16909(8)	16.6233(18)	14.2513(4)
α (°)	90	90	94.902(2)
β (°)	97.0488(4)	98.856(2)	114.365(3)
γ (°)	90	90	109.167(3)
V (Å^3^)	2674.24(2)	2805.0(5)	2062.23(14)
Z	4	4	2
d_calc_ (g cm^−3^)	1.615	1.559	1.312
*μ* (mm^−1^)	5.343	0.610	2.997
F(000)	1342	1360	860
Limiting indices	h = ±10, k = ±26, l = ±20	h = ±10, k = ±25, l = ±20	h = ±15, k = ±17, l = ±17
Reflections coll./uniq.	54351/5622	42783/5515	48563/7978
R(int)	0.0331	0.0238	0.0647
Complete to Θ (%)	100.0	100.0	100.0
Data/restr./param.	5622/0/401	5515/0/380	7978/0/533
GooF on F^2^	1.064	1.058	1.066
Final R indices [I > 2σ(I)]	R_1_ = 0.0309, wR_2_ = 0.0787	R_1_ = 0.0568, wR_2_ = 0.1630	R_1_ = 0.0374, wR_2_ = 0.1016
R indices (all data)	R_1_ = 0.0321, wR_2_ = 0.0798	R_1_ = 0.0621, wR_2_ = 0.1688	R_1_ = 0.0424, wR_2_ = 0.1040
Largest diff. peak/hole (e·Å^−3^)	0.576 and −0.698	0.772 and −0.446	0.408 and −0.321
CCDC no.	2042007	2042006	2042009
