# Supplementary material for: Modulation of Mn3+ Spin State by Guest Molecule Inclusion
Source: Molecules. 2020 Nov 28;25(23):5603. doi: 10.3390/molecules25235603 (PMC7730159; doi:10.3390/molecules25235603)
Supplement: Supplementary file 1 [file molecules-25-05603-s001.pdf]

Supporting Information

Modulation of Mn^{3+} Spin State by Guest Molecule Inclusion

Irina A. Kühne[1], Kane Esien[2], Laurence C. Gavin[1], Helge Müller-Bunz[1], Solveig Felton[2], and Grace G. Morgan[1,*]

Contents

Figure S1. View of asymmetric unit of $[MnL_1]CF_3SO_3{\cdot}0.7H_2O$ at 100 K showing H-bonding connecting complex cation and counterion *via* a water molecule....... 2

Figure S2. View of asymmetric unit of $[MnL_1]CF_3SO_3{\cdot}0.7H_2O$ at 100 K showing H-bonding connecting complex cation and counterion *via* a water molecule....... 3

Figure S3. View of asymmetric unit of $[MnL_1]PF_6{\cdot}0.5H_2O$ at 100 K showing H-bonding between phenoxide oxygen and water molecule. A close contact is formed between the water molecule and the disordered PF_6^- counterion....... 4

Figure S4. View of asymmetric unit of $[MnL_1]PF_6{\cdot}0.5H_2O$ at 293 K showing H-bonding between phenoxide oxygen and water molecule....... 5

Figure S5. View of asymmetric unit of $[MnL_1]PF_6{\cdot}0.3H_2O{\cdot}0.3$sal at 100 K illustrating the absence of intermolecular interactions to the complex cation and showing disorder of partial occupancy 4-methoxysalicylaldehyde guest molecule and water....... 6

Figure S6. Space filling packing arrangement of $[MnL_1]PF_6{\cdot}0.3H_2O{\cdot}0.3$sal at 100 K along the a-axis (left) and along the b-axis (right)....... 7

Figure S7. View of asymmetric unit of $[MnL_1]BPh_4$ at 100 K illustrating the absence of intermolecular interactions....... 8

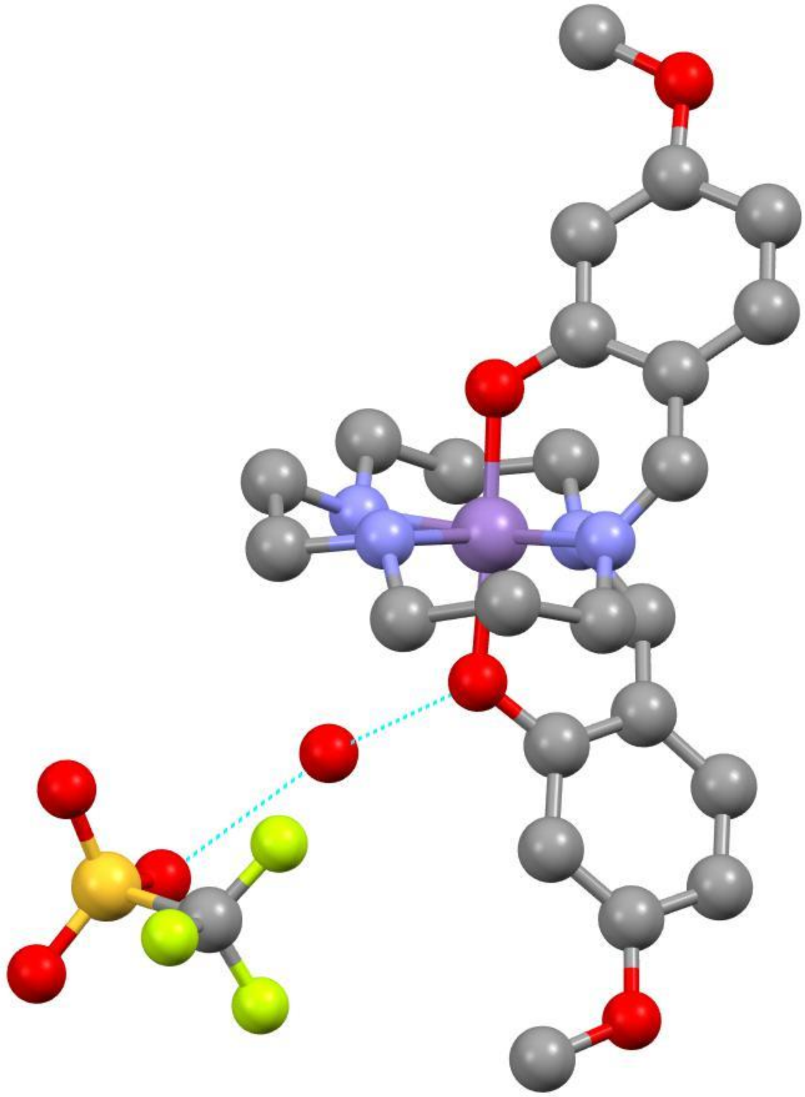

Figure S1. View of asymmetric unit of $[MnL_1]CF_3SO_3{\cdot}0.7H_2O$ at 100 K showing H-bonding connecting complex cation and counterion *via* a water molecule.

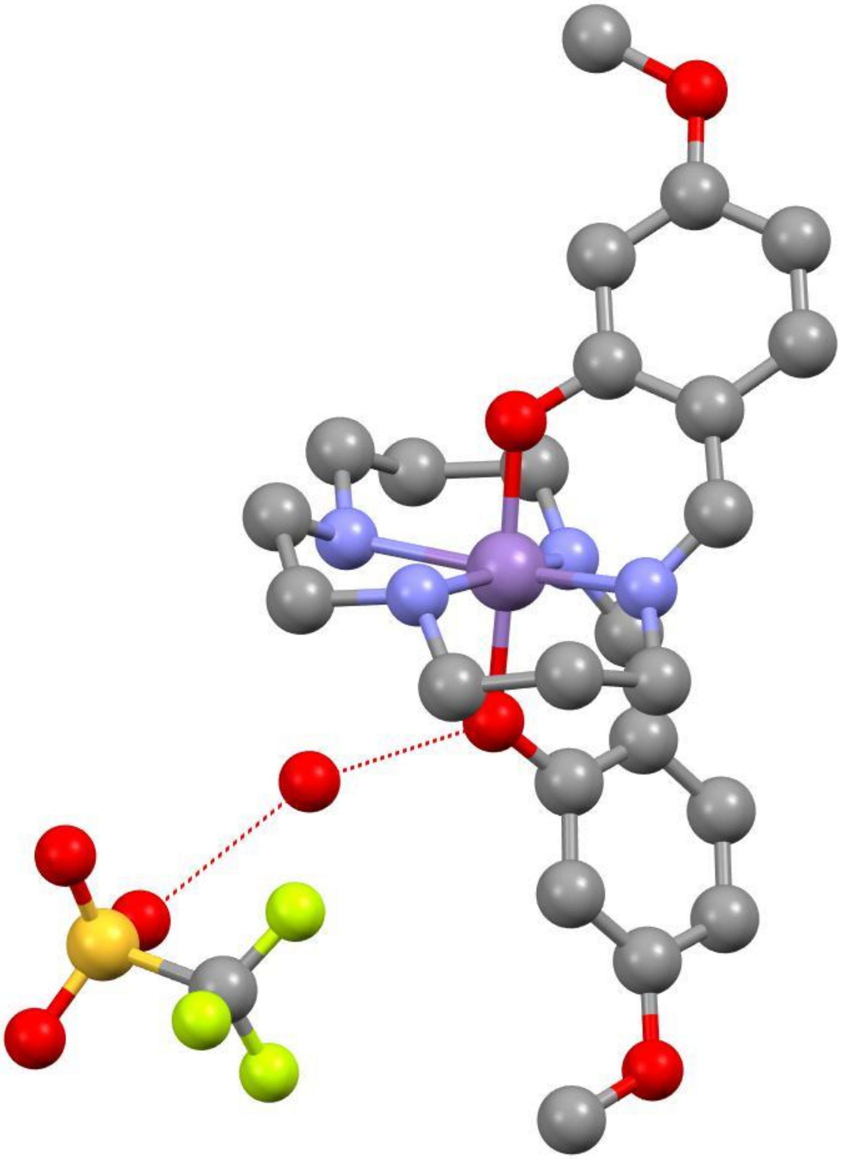

Figure S2. View of asymmetric unit of $[MnL_1]CF_3SO_3{\cdot}0.7H_2O$ at 100 K showing H-bonding connecting complex cation and counterion *via* a water molecule.

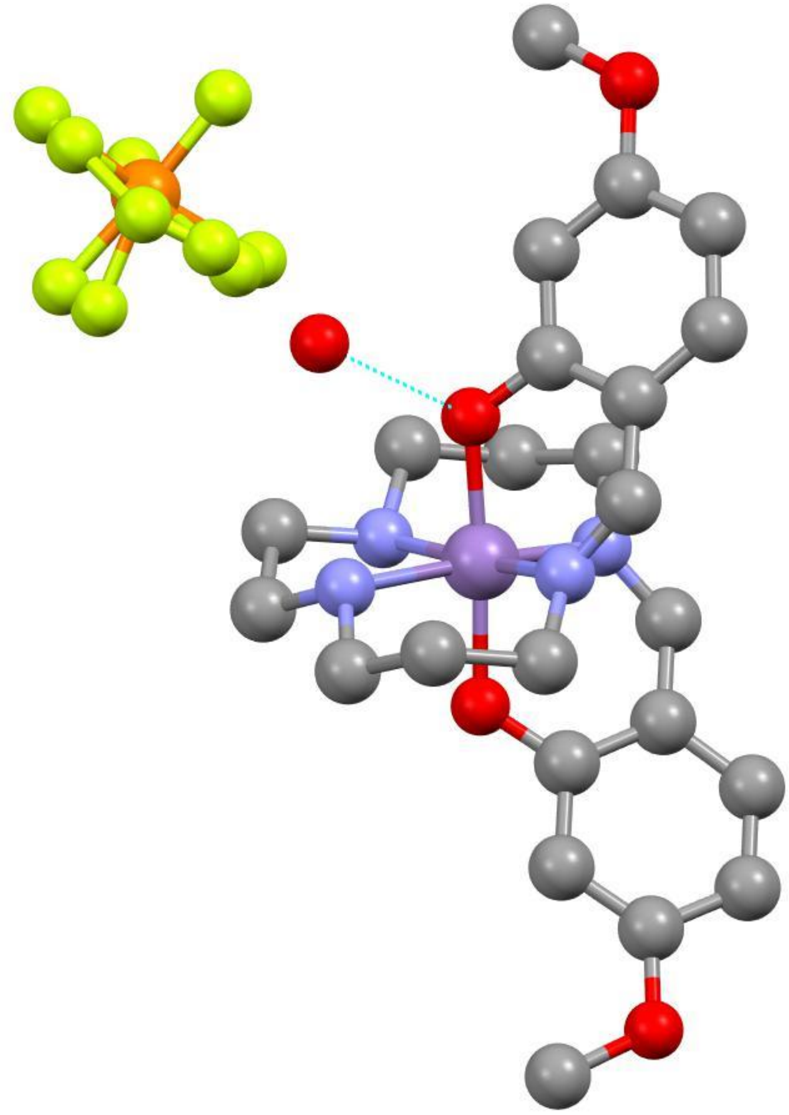

Figure S3. View of asymmetric unit of $[MnL_1]PF_6{\cdot}0.5H_2O$ at 100 K showing H-bonding between phenoxide oxygen and water molecule. A close contact is formed between the water molecule and the disordered PF_6^- counterion.

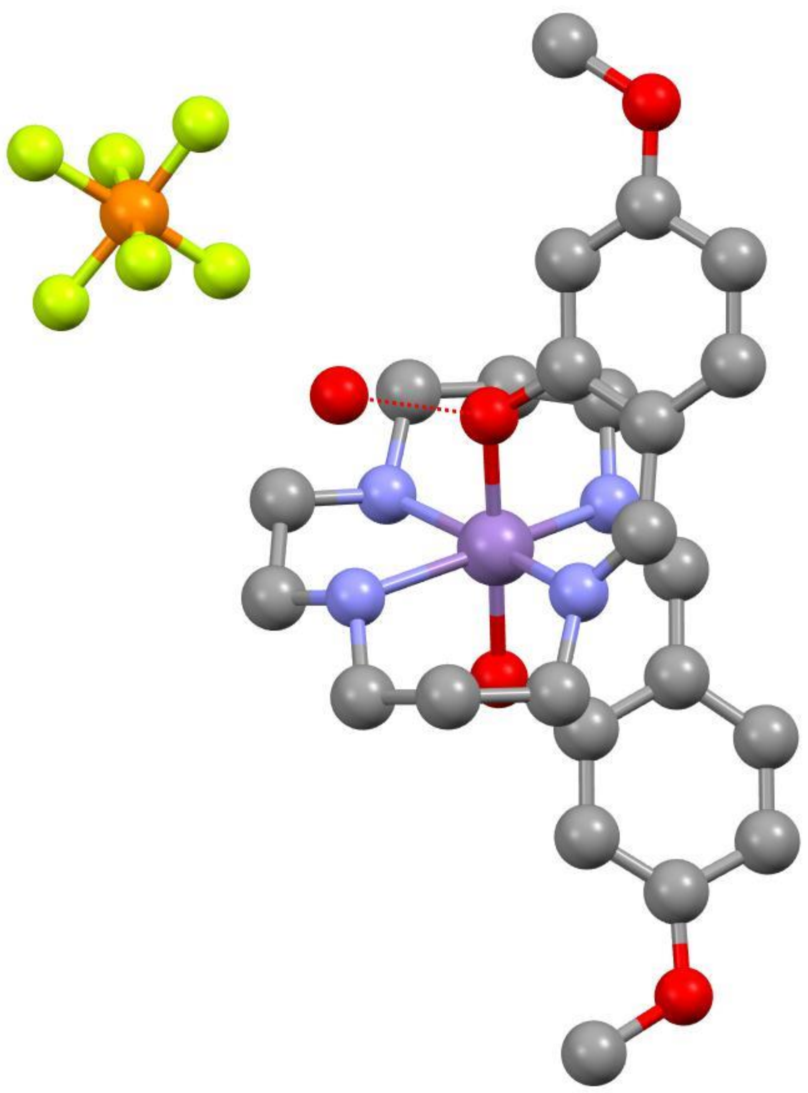

Figure S4. View of asymmetric unit of $[MnL_1]PF_6 \cdot 0.5H_2O$ at 293 K showing H-bonding between phenoxide oxygen and water molecule.

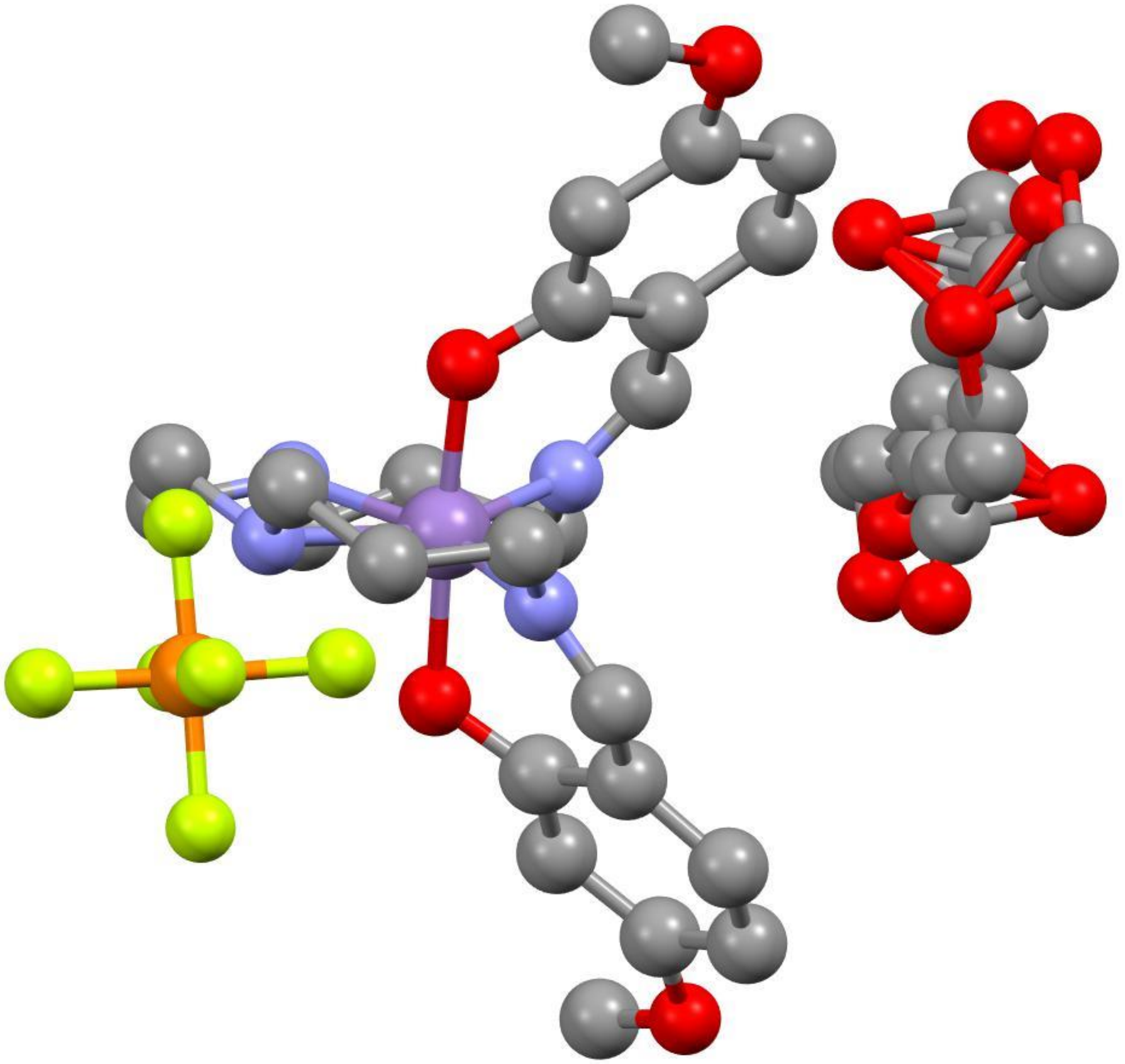

Figure S5. View of asymmetric unit of $[MnL_1]PF_6 \cdot 0.3H_2O \cdot 0.3$sal at 100 K illustrating the absence of intermolecular interactions to the complex cation and showing disorder of partial occupancy 4-methoxysalicylaldehyde guest molecule and water.

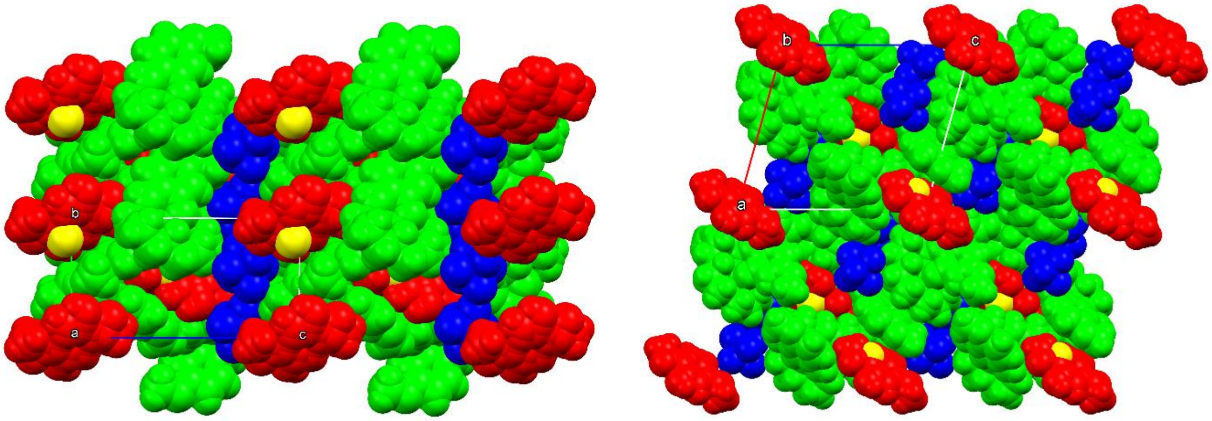

Figure S6. Space filling packing arrangement of $[MnL_1]PF_6{\cdot}0.3H_2O{\cdot}0.3$sal at 100 K along the a-axis (left) and along the b-axis (right) with the $[MnL_1]^+$ in green, PF_6^- in blue, 4-methoxysalicylaldehyde (sal) in red and water molecules in yellow.

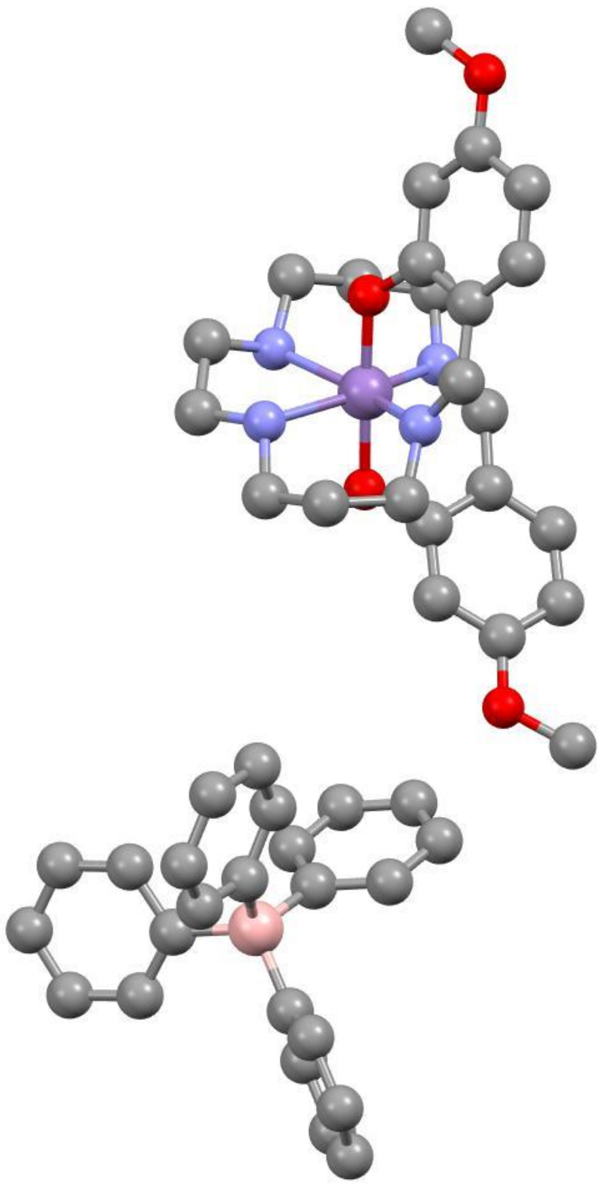

Figure S7. View of asymmetric unit of $[MnL_1]BPh_4$ at 100 K illustrating the absence of intermolecular interactions.
